# Global Hopf Bifurcation on Two-Delays Leslie-Gower Predator-Prey System with a Prey Refuge

**DOI:** 10.1155/2014/619132

**Published:** 2014-04-07

**Authors:** Qingsong Liu, Yiping Lin, Jingnan Cao

**Affiliations:** ^1^Department of Applied Mathematics, Kunming University of Science and Technology, Kunming, Yunnan 650500, China; ^2^School of Computer Science, Beijing University of Post Telecommunication, Beijing 100876, China

## Abstract

A modified Leslie-Gower predator-prey system with two delays is investigated. By choosing *τ*_1_ and *τ*_2_ as bifurcation parameters, we show that the Hopf bifurcations occur when time delay crosses some critical values. Moreover, we derive the equation describing the flow on the center manifold; then we give the formula for determining the direction of the Hopf bifurcation and the stability of bifurcating periodic solutions. Numerical simulations are carried out to illustrate the theoretical results and chaotic behaviors are observed. Finally, using a global Hopf bifurcation theorem for functional differential equations, we show the global existence of the periodic solutions.

## 1. Introduction

The dynamic relationship between predators and their prey has long been and will continue to be one of the dominant topics, not only in ecology but also in mathematical ecology due to its universal existence and importance. In [1], Leslie introduced a predator-prey model in which the “carrying capacity” of the predator's environment is proportional to the number of prey:

(1) x˙=r1x(1−xK)−axy,y˙=r2y(1−yγx),

where *r*_1_, *r*_2_, *K*, and *γ* are positive constants and *x*(*t*) and *y*(*t*) denote the population of the prey and predator at time *t*, respectively. The parameters *r*_1_ and *r*_2_ are the intrinsic growth rates of the prey and the predator. The value *K* is the carrying capacity of the prey, and *γx* takes on the role of a prey-dependent carrying capacity for the predator; the parameter *γ* is a measure of the quality of the prey as food for the predator. However, this model has attracted the attention of some authors [2–4].

Time delays are often incorporated into population models for resource regeneration times, for example, maturing times and gestation periods [5, 6]. Recently, great attention has been received and a lot of work has been carried out on the existence of the Hopf bifurcations in delayed population models (see [7–9] and references cited therein). The stability of positive equilibria and the existence and the direction of the Hopf bifurcations were discussed, respectively, in the references mentioned above. In [10], Yuan and Song considered the following delayed Leslie-Gower predator-prey system:

(2) x˙(t)=r1x(t)(1−x(t−τ)K)−ax(t)y(t),y˙(t)=r2y(t)(1−y(t)γx(t)).

They investigated the stability and the Hopf bifurcation of the above system without considering the effects of time delay on predator.

Motivated by the above discussion, in this paper, by choosing the time delays *τ*_1_ and *τ*_2_ as bifurcation parameters, we investigate a modified Leslie-Gower predator-prey system with two delays described by the following system:

(3) x˙(t)=r1x(t)(1−x(t−τ1)K)−a(1−m)x(t)y(t),y˙(t)=r2y(t)(1−y(t−τ2)γ(1−m)x(t−τ2)),

where *τ*_1_ and *τ*_2_ are all positive constants. Due to crowding, the prey dynamics is delayed by *τ*_1_ [11]. The negative feedback delay *τ*_2_ is assumed in predator growth [12]. *mH* is a refuge protecting of the prey and *m* ∈ [0,1) is a constant. This leaves (1 − *m*)*H* of the prey available to the predator.

The initial conditions for system (3) take the from

(4) $$\begin{array}{c} x\left(\theta \right)=\varphi \left(\theta \right),\;\;\;y\left(\theta \right)=\psi \left(\theta \right),\\ \varphi \left(\theta \right)>0,\;\psi \left(\theta \right)>0,\;\theta \in \left[-\tau ,0\right], \end{array}$$

where (*φ*(*θ*), *ψ*(*θ*))∈([−*τ*, 0], *R*_+0_^2^), *R*_+0_^2^ = {(*x*_1_, *x*_2_) : *x*_*i*_ > 0, *i* = 1,2}.

This paper is organized as follows. In Section 2, we investigate the effect of two delays *τ*_1_ and *τ*_2_ on the stability of the positive equilibrium of system (3). In Section 3, we derive the direction and stability of the Hopf bifurcation by using normal form and central manifold theory. In Section 4, numerical simulations are performed to support the stability results and chaos is observed. Finally, in Section 5, based on the global Hopf bifurcation theorem for general functional differential equations, we investigate the global existence of periodic solutions by using degree theory methods.

## 2. Local Stability Analysis and the Hopf Bifurcation

It is easy to see that system (3) has a unique positive equilibrium *E*_∗_(*x*_∗_, *y*_∗_), where

(5) $$\begin{array}{c} x_{*}=\frac{Kr_{1}}{r_{1}+aK\gamma {\left(1-m\right)}^{2}},\;\;y_{*}=\gamma \left(1-m\right)x_{*}. \end{array}$$

Let $\overset{-}{x}=x-x_{*},\;\;\overset{-}{y}=y-y_{*}$ and still denote by $\overset{-}{x}=x$, $\overset{-}{y}=x$; system (3) can be written as

(6) x˙=α1y(t)+α2x(t−τ1)+α3x(t)x(t−τ1)+α4x(t)y(t),y˙=α5x(t−τ2)+α6y(t−τ2)  +∑i+j+k≥21i!j!k!fijkxi(t−τ2)yj(t−τ2)yk(t),

where

(7) $$\begin{array}{c} \alpha_{1}=-a\left(1-m\right)x_{*},\;\;\alpha_{2}=-\frac{r_{1}x_{*}}{K},\;\;\alpha_{3}=-\frac{r_{1}}{K},\\ \alpha_{4}=-a\left(1-m\right),\;\;\;\alpha_{5}=\gamma \left(1-m\right)r_{2},\;\;\alpha_{6}=-r_{2},\\ f=r_{2}y_{1}\left(1-\frac{y}{\gamma \left(1-m\right)x}\right),\;\;{f_{ijk}=\frac{\partial^{i+j+k}f^{\left(1\right)}}{\partial x^{i}\partial y^{j}\partial k^{k}}|}_{\left(x_{*},y_{*},y_{*}\right)}. \end{array}$$

We then obtain the linearized system

(8) x˙=α1y(t)+α2x(t−τ1),y˙=α5x(t−τ2)+α6y(t−τ2).

The corresponding characteristic equation is

(9) $$\begin{array}{c} \lambda^{2}-\left(A\lambda +B\right)e^{-\lambda \tau_{2}}-C\lambda e^{-\lambda \tau_{1}}+Ee^{-\lambda (\tau_{1}+\tau_{2})}=0, \end{array}$$

where

(10) $$\begin{array}{c} A=\alpha_{6},\;\;B=\alpha_{1}\alpha_{5},\;\;C=\alpha_{2},\;\;\;E=\alpha_{2}\alpha_{6}. \end{array}$$

**Case 1**

For  *τ*_1_ = *τ*_2_ = 0, (9) becomes

(11) $$\begin{array}{c} \lambda^{2}-\left(A+C\right)\lambda -B+E=0. \end{array}$$

Since *A* + *C* < 0, −*B* + *E* > 0, we know that all roots have negative real parts.

**Theorem 1**

For *τ*_1_ = *τ*_2_ = 0, the interior equilibrium point (*x*_∗_, *y*_∗_) is locally asymptotically stable.

**Case 2**

Consider

(12) $$\begin{array}{c} \tau_{1}=0,\;\tau_{2}>0. \end{array}$$

**Theorem 2**

For *τ*_1_ = 0, the interior equilibrium point *E*_∗_ = (*x*_∗_, *y*_∗_) is locally asymptotically stable for 0 < *τ*_2_ < *τ*_2_0__ and it undergoes the Hopf bifurcation at *τ*_2_ = *τ*_2_0__ given by

(13) τ20=1ω20
arccos
[(E−B−AC)ω102A2ω202+(B−E)2].

**Proof**

On substituting *τ*_1_ = 0, the characteristic equation (9) becomes

(14) $$\begin{array}{c} \lambda^{2}-C\lambda -\left(A\lambda +B-E\right)e^{-\lambda \tau_{2}}=0. \end{array}$$

Let *iω* (*ω* > 0) be a purely imaginary root of (14); then it follows that

(15) $$\begin{array}{c} A\omega \sin\omega \tau_{2}+\left(B-E\right)\cos\omega \tau_{2}=-\omega^{2},\\ A\omega \cos\omega \tau_{2}-\left(B-E\right)\sin\omega \tau_{2}=-C\omega . \end{array}$$

Squaring both sides and adding them up, we get the following polynomial equation:

(16) $$\begin{array}{c} \omega^{4}+\left(C^{2}-A^{2}\right)\omega^{2}-{\left(B-E\right)}^{2}=0. \end{array}$$

It is easy to know that (16) has unique positive root *ω*_2_0__^2^; then the corresponding critical value of time delay *τ*_2_*n*__ is

(17) τ2n=1ω20arccos[(E−B−AC)ω202A2ω202+(B−E)2]+2nπω20,n=0,1,2,….

Let *λ*(*τ*_2_*n*__) = ±*iω*_2_0__ be the root of (14); then the transversal condition can be obtained:

(18) $$\begin{array}{c} {\left(\frac{d\lambda }{d\tau_{2}}\right)}_{\tau_{2}=\tau_{2_{n}}}^{-1}=\frac{\left(C-2\lambda \right)e^{\lambda \tau_{2}}}{\lambda \left(A\lambda +B-E\right)}+\frac{A}{\lambda \left(A\lambda +B-E\right)}-\frac{\tau_{2}}{\lambda }. \end{array}$$

Since

(19) Sign{d(Reλ(τ2))dτ2}τ2=τ2n−1 =Sign{[Re(C−2λ)eλτ2λ(Aλ+B−E)]τ2=τ2n      +[ReAλ(Aλ+B−E)]τ2=τ2n},

we can obtain

(20) Sign{d(Reλ(τ2))dτ2}τ2=τ2n−1 =Sign{Re[Ccos⁡⁡ω20τ2+2ω20sin⁡ω20τ2−Aω202+i(B−E)ω20]+Re[iCsin⁡ω20τ2−2ω20cos⁡⁡ω20τ2−Aω202+i(B−E)ω20]+Re[C−Aω202+i(B−E)ω20]} =Sign{(C2−A2)+2ω202A2ω204+(B−E)2ω202} =Sign(C2−A2)2+4(B−E)2A2ω202+(B−E)2>0,

and then we can obtain

(21) d(Reλ)dτ2|τ2=τ20>0.

**Case 3**

Consider

(22) $$\begin{array}{c} \tau_{2}=0,\;\tau_{1}>0. \end{array}$$

**Theorem 3**

If *τ*_2_ = 0 holds, the interior equilibrium point *E*_∗_(*x*_∗_, *y*_∗_) is locally asymptotically stable for 0 < *τ*_1_ < *τ*_1_0__ and it undergoes the Hopf bifurcation at *τ*_1_ = *τ*_1_0__ given by

(23) τ10=1ω10
arccos
((−ω102+B)E−ACω102E2+C2ω102),

where *iω*_1_0__ is root of the corresponding characteristic equation.

**Proof**

The proof is similar to that in Case 2.

**Case 4**

*τ*_2_ is fixed in the interval (0, *τ*_2__0_) and *τ*_1_ > 0.

**Theorem 4**

Assume that *B* + *E* > 0 and *τ*_2_ ∈ (0, *τ*_2_0__); then the equilibrium *E*_∗_(*x*_∗_, *y*_∗_) is asymptotically stable for *τ*_1_ ∈ (0, *τ*_1_0__′); moreover let ((*H* _1_)) hold; ((*H* _1_)) is defined below; then system (3) undergoes the Hopf bifurcation at *E*_∗_(*x*_∗_, *y*_∗_) when *τ*_1_ = *τ*_1_0__′, where

(24) $$\begin{array}{c} \tau_{1_{0}}^{\prime }=\frac{1}{\omega_{*}}\text{ arccos }\left[\frac{E_{2}\left(k_{2}-k_{3}\right)-E_{1}\left(k_{1}-k_{2}-k_{3}\right)}{E_{1}^{2}+E_{2}^{2}}\right]. \end{array}$$

**Proof**

We know *τ*_2_ in its stable interval and *τ*_1_ is considered as a parameter. Let *iω* (*ω* > 0) be a root of (9). Separating real and imaginary parts, leads to

(25) −ω2−Bcos⁡ωτ2−(A+C)ωsinωτ2  =−Ecos⁡ωτ2cos⁡ωτ1+Esinωτ2sinωτ1,Bsinωτ2−(A+C)ωcos⁡ωτ2  =Ecos⁡ωτ2sinωτ1+Esinωτ2cos⁡ωτ1.

Let

(26) $$\begin{array}{c} H\left(\omega \right)=\omega^{4}+l_{1}\omega^{3}+l_{2}\omega^{2}+l_{3}=0, \end{array}$$

where

(27) l1=2(A+C)sinωτ2,l2=B+A+2AC+C2,l3=B2−E2.

We assumed that

(28) $$\begin{array}{c} B+E>0. \end{array}$$

Then *H*(0) < 0 and *H*(*∞*) = *∞*.

With going detailed analysis (26) it is assumed that there exists at least one real positive root *ω*_∗_. Now (25) can be written as

(29) $$\begin{array}{c} k_{1}-k_{2}-k_{3}=-E_{1}\cos\omega_{*}\tau_{1}+E_{2}\sin\omega_{*}\tau_{1},\\ k_{4}-k_{5}=E_{1}\sin\omega_{*}\tau_{1}+E_{2}\cos\omega_{*}\tau_{1}, \end{array}$$

where

(30) $$\begin{array}{c} k_{1}=-\omega_{*}^{2},\;\;k_{2}=B\cos\omega_{*}\tau_{2},\\ k_{3}=\left(A+C\right)\omega_{*}\sin\omega_{*}\tau_{2},\;\;k_{4}=B\sin\omega_{*}\tau_{2},\\ k_{5}=\left(A+C\right)\omega_{*}\cos\omega_{*}\tau_{2},\;\;E_{1}=E\cos\omega_{*}\tau_{2},\\ E_{2}=E\sin\omega_{*}\tau_{2}. \end{array}$$

Equation (29) is simplified to give

(31) τ1j′=1ω∗arccos[E2(k4−k5)−E1(k1−k2−k3)E12+E22]+2jπω∗,j=0,1,2,…,

and ±*iω*_∗_ are purely imaginary roots of (9) for *τ*_2_ ∈ (0, *τ*_2_0__]. Now verify the transversal condition of the Hopf bifurcation; differentiating equation (9) with respect to *τ*_1_, it is obtained that

(32) (dλdτ1)τ1=τ1j′−1=(2λ−Ae−λτ2+(Aλ+B)τ2e−λτ2−Ce−λτ1j′  +Cλτ1j′e−λτ1j′−(τ1j′+τ2)Ee−λ(τ1j′+τ2)) ×(Eλe−λ(τ1j′+τ2)−Cλ2τ1j′e−λτ1j′)−1=P1+iQ1M1+iN1,

where

(33) P1=−Acos⁡ω∗τ2−Aω∗τ2sinω∗τ2+Bτ2cos⁡ω∗τ2    −Ccos⁡ω∗τ1j′+Cω∗τ1j′sinω∗τ1j′    −E(τ1j′+τ2)cos⁡ω∗(τ1j′+τ2),Q1=2ω∗+Asinω∗τ2−Aω∗τ2cos⁡ω∗τ2−Bτ2sinω∗τ2  +Csinω∗τ1j′+Cω∗τ1j′cos⁡ω∗τ1j′  +E(τ1j′+τ2)sinω∗(τ1j′+τ2),M1=Eω∗sinω∗(τ1j′+τ2)+Cω∗2cos⁡ω∗τ1j′,N1=Eω∗cos⁡ω∗(τ1j′+τ2)−Cω∗2sinω∗τ1j′.

Then

(34) (M12+N12)[Re(dλdτ1)]τ1=τ1j′−1=M1P1+N1Q1,

noting that

(35) Sign[Re(dλdτ1)]|τ1=τ10′=Sign[Re(dλdτ1)−1]|τ1=τ10′.

To obtain the transversal condition, we also need the condition as follows:

$$\begin{array}{cc} \left(H_{1}\right) & M_{1}P_{1}+N_{1}Q_{1}\neq 0. \end{array}$$

**Case 5**

*τ*_1_ is fixed in the interval (0, *τ*_1_0__) and *τ*_2_ > 0.

**Theorem 5**

Assume that ((*H* _2_)) holds; let *B* + *E* > 0 and *τ*_1_ ∈ (0, *τ*_1_0__); then the equilibrium *E*_∗_(*x*_∗_, *y*_∗_) is asymptotically stable for *τ*_2_ ∈ (0, *τ*_2_0__′), and system (3) undergoes the Hopf bifurcation at *E*_∗_(*x*_∗_, *y*_∗_) when *τ*_2_ = *τ*_2_0__′, where

(36) τ20′=1ω0
arccos
[(−Cω0cos⁡ω0τ1(Aω0+Esinω0τ1)       −(B−Ecos⁡ω0τ1)       ×(ω02+Cω0sinω0τ1))      ×((Aω0+Esinω0τ1)2      ×(B−Ecos⁡ω0τ1)2)−1],

$$\begin{array}{cc} \left(H_{2}\right) & {\;M}_{2}P_{2}+N_{2}Q_{2}\neq 0 \end{array}$$

and *iω*_0_ (*ω*_0_ > 0) is root of the corresponding characteristic equation; moreover

(37) P2=−Acos⁡ω0τ2+Aω0τ2sinω0τ2+Bτ2cos⁡ω0τ2    −Ccos⁡ω0τ1+Cω0τ1sinω0τ1    −E(τ1+τ2)cos⁡ω0(τ1+τ2),Q2=2ω0+Asinω0τ2+Aω0τ2cos⁡ω0τ2−Bτ2sinω0τ2   +Csinω0τ1+Cω0τ1cos⁡ω0τ1   +E(τ1+τ2)sinω0(τ1+τ2),M2=Eω0sinω0(τ1+τ2)+Aω02cos⁡ω0τ1−Bω0sinω0τ2,N2=Eω0cos⁡ω0(τ1+τ2)−Aω02sinω∗τ1−Bω0cos⁡ω0τ2.

**Proof**

The proof is similar to that in Case 4.

## 3. Direction and Stability of the Hopf Bifurcation

In this section, we show that the system undergoes the Hopf bifurcation for different combinations of *τ*_1_ and *τ*_2_ satisfying sufficient conditions as described. Using the method based on the normal form theory and center manifold theory introduced by Hassard et al. in [13], we study the direction of bifurcations and the stability of bifurcating periodic solutions. Throughout this section, it is considered that the system undergoes the Hopf bifurcation at *τ*_2_ = *τ*_2_0__′, *τ*_1_ ∈ (0, *τ*_1_0__) at *E*_∗_. Let *τ*_2_ = *τ*_2_0__′ + *μ*, *μ* ∈ *R*, so that the Hopf bifurcation occurs at *μ* = 0. Without loss of generality, it is assumed that *τ*_1_* < *τ*_2_0__′ where *τ*_1_* ∈ (0, *τ*_1_0__). Now we rescale the time by *t* = *tτ*_2_, *X*(*t*) = *x* − *x*_∗_, *Y*(*t*) = *y* − *y*_∗_; then system (3) can be written as

(38) U˙(t)=(τ20′+μ)(B1U(t)+B2U(t−τ1∗τ2)      +B3U(t−1)+f(x,y)),

where

(39) $$\begin{array}{c} U\left(t\right)={\left(X\left(t\right),Y\left(t\right)\right)}^{T},\\ B_{1}=\left(\begin{array}{cc} 0 & \alpha_{1}\\ 0 & 0 \end{array}\right),\;\;B_{2}=\left(\begin{array}{cc} \alpha_{2} & 0\\ 0 & 0 \end{array}\right),\\ B_{3}=\left(\begin{array}{cc} 0 & 0\\ \alpha_{5} & \alpha_{6} \end{array}\right),\;\;f={\left(f_{1},f_{2}\right)}^{T}. \end{array}$$

For convenience, *X*(*t*), *Y*(*t*) are still as *x*(*t*), *y*(*t*), respectively; the nonlinear terms *f*_1_ and *f*_2_ are

(40) f1=α3x(t)x(t−τ1∗τ2)+α4x(t)y(t),f2=∑i+j+k≥21i!j!k!fijkxi(t−1)yj(t−1)yk(t).

Define a family of operators as

(41) Lμφ=(τ20′+μ)(B1φ(0)+B2φ(−τ1∗τ2)+B3φ(−1)), φ=(φ1,φ2)T∈C([−1,0],R2).

By the Riesz representation theorem, there exists a matrix whose components are bounded variation functions *η*(*θ*, *μ*):[−1,0] → *R* ^2^ such that

(42) $$\begin{array}{c} L_{\mu }\varphi =\overset{0}{\underset{-1}{\int }}d\eta \left(\theta ,\mu \right)\varphi \left(\theta \right), \end{array}$$

where we choose

(43) $$\begin{array}{c} \eta \left(\theta ,\mu \right)=\{\begin{array}{cc} \left(\tau_{2_{0}}^{\prime }+\mu \right)\left(B_{1}+B_{2}+B_{3}\right), & \theta =0,\\ \left(\tau_{2_{0}}^{\prime }+\mu \right)\left(B_{2}+B_{3}\right), & \theta \in [-\frac{\tau_{1}^{*}}{\tau_{2}},0),\\ \left(\tau_{2_{0}}^{\prime }+\mu \right)B_{3}, & \theta \in \left(-1,-\frac{\tau_{1}^{*}}{\tau_{2}}\right),\\ 0, & \theta =-1. \end{array} \end{array}$$

For *φ* = (*φ*_1_, *ϕ*_2_)^*T*^ ∈ *C*([−1,0], *R* ^2^), define

(44) A(μ)φ={dφ(θ)dθ,θ∈[−1,0),∫−10dη(s,μ)φ(s),θ=0,R(μ)φ={0,θ∈[−1,0),h(μ,φ),θ=0,

where

(45) h(μ,φ)=(τ20′+μ)(h1h2),φ=(φ1,φ2)T∈C([−1,0],R2),h1=α3φ1(0)φ1(−τ1∗τ2)+α4φ1(0)φ2(0),h2=∑i+j+k≥21i!j!k!fijkφ1i(−1)φ2j(−1)φ2k(0).

Hence, (3) can be rewritten as

(46) $$\begin{array}{c} {\dot{U}}_{t}=A\left(\mu \right)U_{t}+R\left(\mu \right)U_{t}, \end{array}$$

where *U* = (*X*(*t*), *Y*(*t*))^*T*^ and *U*_*t*_(*θ*) = *U*(*t* + *θ*), *θ* ∈ [−1,0]. For *ψ* ∈ *C*([0,1], (*R* ^2^)*), define *A*(0) = *A* and the adjoint operator A* of *A* as

(47) $$\begin{array}{c} A^{*}\psi \left(s\right)=\{\begin{array}{cc} -\frac{d\psi \left(s\right)}{ds}, & s\in \left(0,1\right],\\ \overset{0}{\underset{-1}{\int }}d\eta^{T}\left(t,0\right)\psi \left(-t\right), & s=0, \end{array} \end{array}$$

where *η* ^*T*^ is the transpose of the matrix *η*.

For *φ* ∈ *C*([−1,0], *R* ^2^) and *ψ* ∈ *C*([0,1], (*R* ^2^)*), in order to normalize the eigenvectors of operator *A* and adjoint operator A*, we define a bilinear inner product

(48) 〈ψ(s),φ(θ)〉=ψ−(0)φ(0) −∫−10∫ξ=0θψ−(ξ−θ)dη(θ)φ(ξ)dξ,

where *η*(*θ*) = *η*(*θ*, 0).

Since ±*iω*_0_ *τ*_2_0__′ are eigenvalues of *A*, they will also be the eigenvalues of A*. The eigenvectors of *A* and A* are calculated corresponding to the eigenvalues +*iω*_0_ *τ*_2_0__′ and −*iω*_0_ *τ*_2_0__′.

**Lemma**

*q*(*θ*) = (1,*ρ*)^*T*^ *e* ^*iω*_0_*τ*_2_0__′*θ*^ is the eigenvector of *A* corresponding to +*iω*_0_ *τ*_2_0__′; q*(*s*) = (1/*D*)(1,*σ*)^*T*^ *e* ^*iω*_0_*τ*_2_0__′*s*^ is the eigenvector of A* corresponding to −*iω*_0_ *τ*_2_0__′ and

(49) $$\begin{array}{c} \langle q^{*}\left(s\right),q\left(\theta \right)\rangle =1,\;\;\langle q^{*}\left(s\right),\overset{-}{q}\left(\theta \right)\rangle =0, \end{array}$$

where

(50) ρ=iω0−α2e−iω0(τ1∗/τ20′)α1,  σ=−iω0+α2e−iω0τ20′α5eiω0τ20′,D−=1+ρσ−+τ20′[(α5σ−+α6ρσ−)e−iω0τ20′+τ1∗τ20′α2e−iω0(τ1∗/τ20′)].

Following the algorithms explained in Hassard et al. [13], we can obtain the properties of the Hopf bifurcation:

(51) g20=2τ20′D−[α3e−iω0(τ1∗/τ20′)+α4ρ   +σ−(12f200e−2iω0τ20′+f110ρe−2iω0τ20′      +f101ρe−2iω0τ20′+f011ρ2e−iω0τ20′)],g11=τ20′D−[2α3e−iω0(τ1∗/τ20′)+α4(ρ−+ρ)+σ−(f200+f110(ρ−+ρ)+f101ρeiω0τ20′+f101ρ−e−iω0τ20′+2f011ρ−ρe−iω0τ20′)],g02=2τ20′D−[α3e−iω0(τ1∗/τ20′)+α4(ρ−+ρ)     +σ−(12f200e2iω0τ20′+f110ρ−e2iω0τ20′ +f101ρ−eiω0τ20′+f011ρ−2ρeiω0τ20′)],g21=2τ20′D−{α3(W11(1)(τ1∗τ20′)+12W20(1)(τ1∗τ20′)         +12W20(1)(0)e−iω0(τ1∗/τ20′)         +W11(1)(0)e−iω0(τ1∗/τ20′))     +α4(W11(2)(0)+12W20(2)(0)         +12W20(1)(0)ρ−+ρW11(1)(0))     +σ−[12f200(2W11(1)(−1)e−iω0τ20′            +W20(1)(−1)eiω0τ20′)       +f110(W11(1)(−1)e−iω0τ20′            +12W20(2)(−1)eiω0τ20′            +12W20(1)(−1)ρ−eiω0τ20′             +W11(1)(−1)ρe−iω0τ20′)       +f101(W11(1)(−1)ρ+12W20(1)(−1)ρ−            +12W20(2)(0)eiω0τ20′            +W11(2)(0)e−iω0τ20′)       +f011(W11(2)(−1)ρ+12W20(2)(−1)ρ−            +12W20(2)(0)ρ−eiω0τ20′            +W11(2)(0)ρe−iω0τ20′            +12f300eiω0τ20′)]},

where

(52) W20(θ)=ig20ω0τ20′q(0)eiθω0τ20′     +ig−023τ20′ω0q−(0)e−iθω0τ20′+ Re2iθω0τ20′, W11(θ)=−ig11τ20′ω0q(0)eiθω0τ20′     +ig−11τ20′ω0q−(0)e−iθω0τ20′+S.

We know that *R* = (*R* ^(1)^, *R* ^(2)^) ∈ *R* ^2^ and *S* = (*S* ^(1)^, *S* ^(2)^) ∈ *R* ^2^ are constant vectors, computed as

(53) R=2(2iω0−α2e−2iω0(τ1∗/τ20′)−α1−α5e−2iω0τ20′2iω0−α6e−2iω0τ20′)−1 ×(α3e−iω0(τ1∗/τ20′)+α4ρ(12f200+f110ρ+f101ρ)e−2iω0τ20′+f011ρ2e−iω0τ20′),S=(−α2−α1−α5−α6)−1 ×(2α3e−iω0(τ1∗/τ20′)+α4(ρ−+ρ)f200+f110(ρ−+ρ)+f101ρeiω0τ20′+(f101ρ−+2f011ρ−ρ)e−iω0τ20′).

As a result, we know *W*_20_(*θ*) and *W*_11_(*θ*); then *g*_*ij*_ is determined by the parameters and delays *τ*_2_0__′ and *τ*_1_*. Thus, we can compute the following quantities:

(54) c1(0)=i2ω0τ20′(g20g11−2|g11|2−13|g02|2)+g212,μ2=−Re{c1(0)}Re{λ′(τ20′)},β2=2Re{c1(0)},T2=−Im⁡{c1(0)}+μ2Im⁡{λ′(τ20′)}ω0τ20′.

These expressions give a description of the bifurcating periodic solutions in the center manifold of system (3) at critical values *τ*_2_ = *τ*_2_0__′ and when *Re*{*λ*′(*τ*_2_0__′)} > 0 which can be stated as follows:

- *μ*_2_ gives the direction of the Hopf bifurcation: if *μ*_2_ > 0  (*μ*_2_ < 0), the Hopf bifurcation is supercritical (subcritical);
- *β*_2_ determines the stability of bifurcating periodic solution: the periodic solutions are stable (unstable) if *β*_2_ < 0  (*β*_2_ > 0);
- *T*_2_ denotes the period of bifurcating period solutions: if *T*_2_ > 0  (*T*_2_ < 0), periodic solutions increase (decrease).

## 4. Numerical Simulations

To demonstrate the algorithm for determining the existence of the Hopf bifurcation in Section 2 and the direction and stability of the Hopf bifurcation in Section 3, we carry out numerical simulations on a particular case of (3) in the following form:

(55) x˙(t)=0.8x(t)(1−x(t−τ1)0.7)−1.3(1−0.5)x(t)y(t),y˙(t)=y(t)(1−y(t−τ2)(1−0.5)x(t−τ2)),

where *r*_1_ = 0.8, *r*_2_ = 1, *a* = 1.3, *K* = 0.7, *γ* = 1, and *m* = 0.5. It is easy to show that system (55) has unique coexistence equilibrium *E*_∗_(0.545,0.2725). By calculation, when *τ*_1_ = 0, the critical delay for *τ*_2_ is obtained as *τ*_2_0__ = 1.3507 and *τ*_1_0__ = 5.8228 when *τ*_2_ = 0.

**Figure 1:**
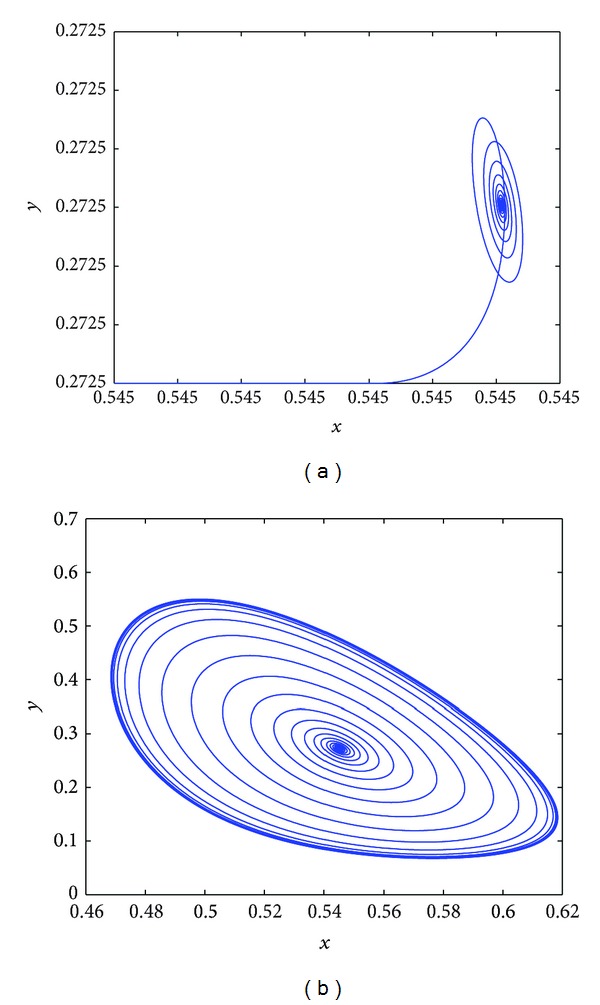
(a) *E*_∗_ is asymptotically stable equilibrium at *τ*_1_ = 0 and *τ*_2_ = 1.1; (b) *E*_∗_ loses stability and the Hopf bifurcation occurs at *τ*_1_ = 0, *τ*_2_ = 1.5.

**Figure 2:**
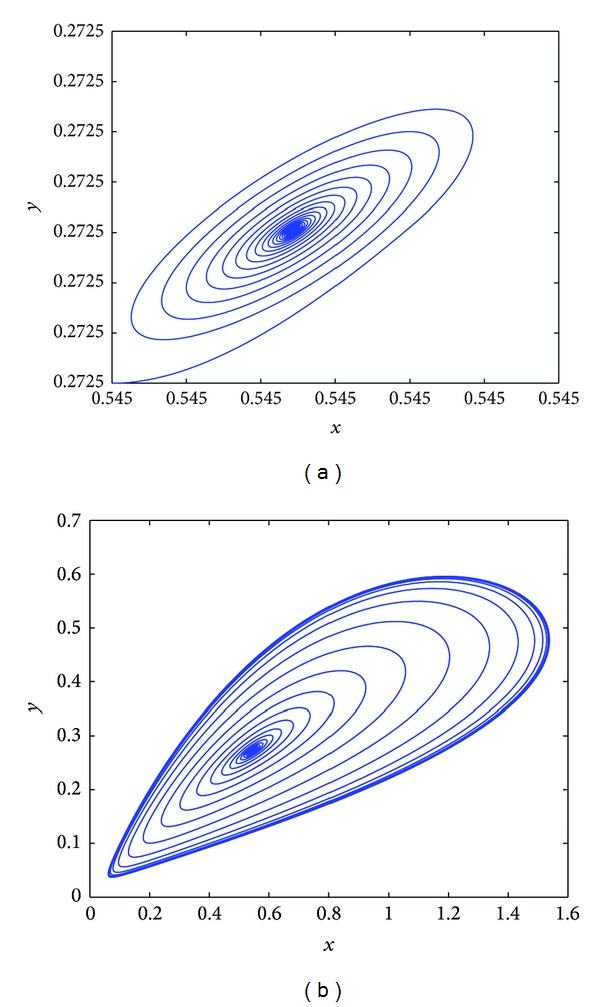
(a) *E*_∗_ is asymptotically stable equilibrium at *τ*_1_ = 2.8 and *τ*_2_ = 0; (b) *E*_∗_ loses stability and the Hopf bifurcation occurs at *τ*_1_ = 6.5, *τ*_2_ = 0.

We can see from Figure 1(a) that *E*_∗_ is asymptotically stable at *τ*_1_ = 0, *τ*_2_ = 1.1 < *τ*_2_0__ = 1.3507, while from Figure 1(b) *E*_∗_ loses stability and the Hopf bifurcation occurs at *τ*_1_ = 0, *τ*_2_ = 1.5 > *τ*_2_0__ = 1.3507. From Figure 2(a), *E*_∗_ is asymptotically stable when *τ*_1_ = 2.8 < *τ*_1_0__ = 5.8228, *τ*_2_ = 0, while from Figure 2(b) *E*_∗_ loses stability and the Hopf bifurcation occurs when *τ*_1_ = 6.5 > *τ*_1_0__ = 5.8228, *τ*_2_ = 0.

**Figure 3:**
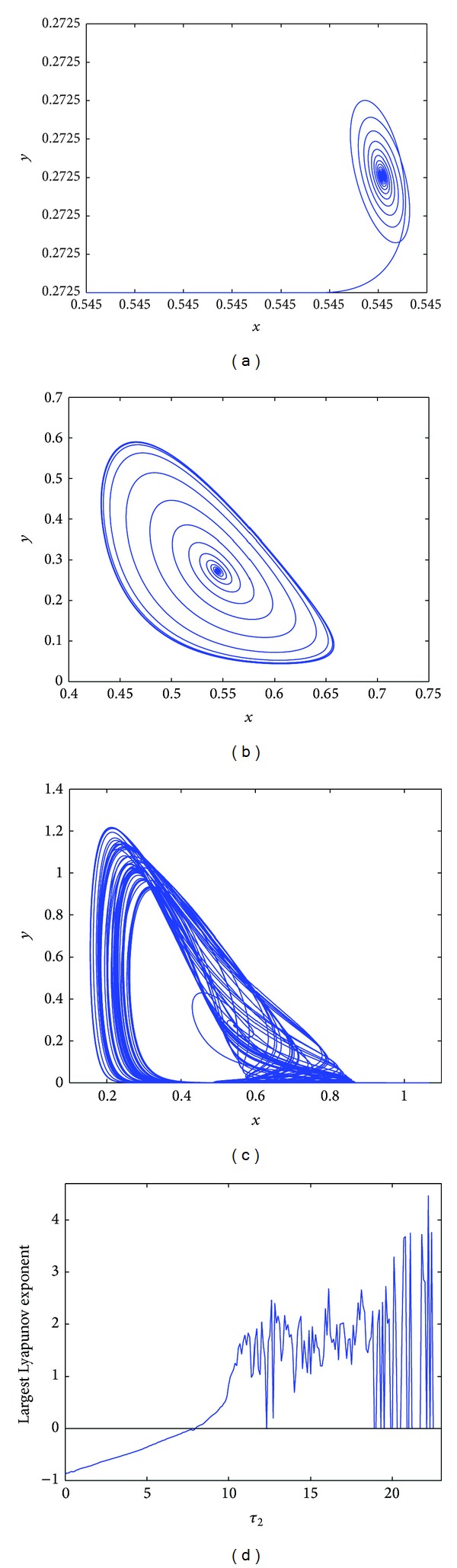
Under the condition of *τ*_1_ = 1.28, (a) *E*_∗_ is asymptotically stable equilibrium at *τ*_2_ = 1.32; (b) *E*_∗_ loses stability and the Hopf bifurcation occurs at *τ*_2_ = 5.83; (c) a chaotic solution occurs at *τ*_2_ = 7.9; (d) the largest Lyapunov exponent diagram of system (55) for variable *τ*_2_.

Further, under the condition of *τ*_1_ = 1.28, when *τ*_2_ = 1.32 < *τ*_2_0__′ = 1.9507, *E*_∗_ is also stable (see Figure 3(a)), while, at *τ*_2_ = 5.83, *E*_∗_ loses stability and the Hopf bifurcation occurs from Figure 3(b); then using the algorithm derived in Section 3, we obtain that *μ*_2_ = 312.8, *β*_2_ = −287.5, *T*_2_ = 106.56; we know the Hopf bifurcation is supercritical and bifurcating periodic solutions are stable and increase. When *τ*_2_ = 7.9, system (55) becomes a chaotic solution in Figure 3(c). In Figure 3(d), the largest Lyapunov exponent diagram is plotted for variable *τ*_2_; it is easy to know that when *τ*_2_ > 7.55, the Lyapunov exponent is almost positive; then the chaos occurs.

**Figure 4:**
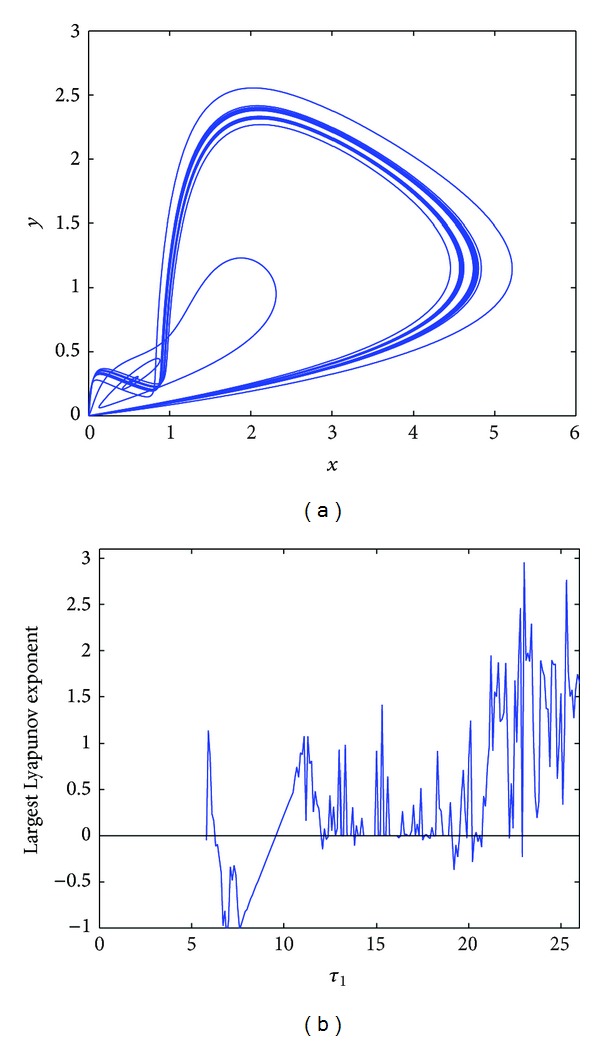
(a) A chaotic solution occurs at *τ*_1_ = 10.15, *τ*_2_ = 1.2; (b) the largest Lyapunov exponent diagram of system (55) for variable *τ*_1_.

Whereas, when *τ*_1_ = 10.15 > *τ*_1_0__ = 5.8228 and *τ*_2_ = 1.2, system (55) becomes chaotic in Figure 4(a), in Figure 4(b), the largest Lyapunov exponent diagram is plotted for variable *τ*_1_; it is easy to know that when *τ*_1_ > 9.85, the Lyapunov exponent is almost positive; then the chaotic solutions occur.

**Figure 5:**
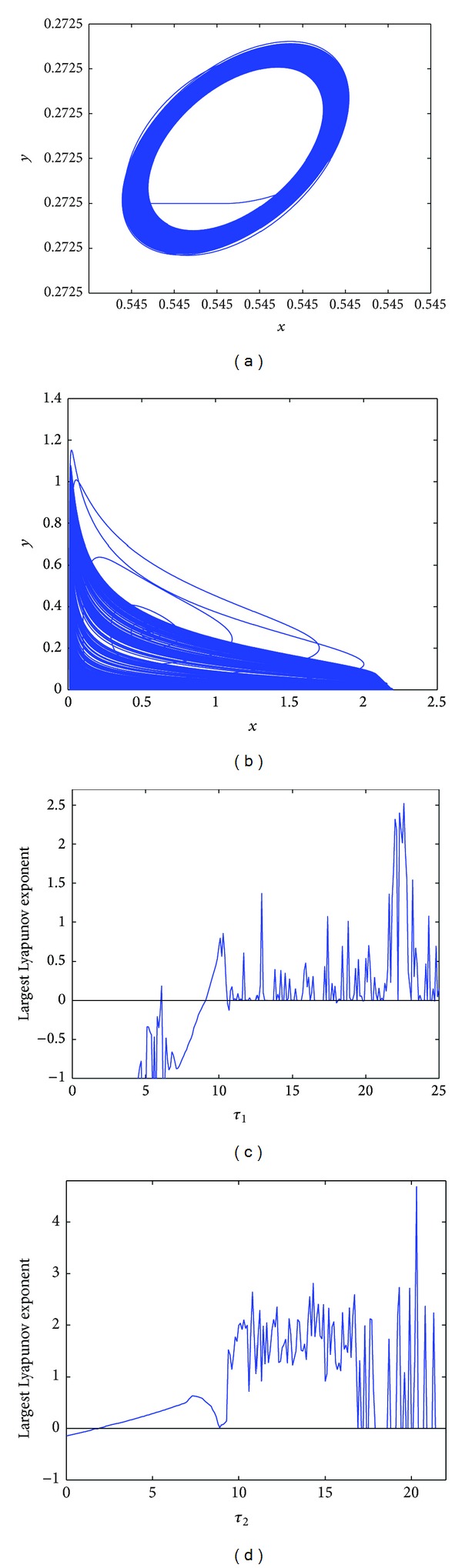
(a) The Hopf bifurcation occurs at *τ*_1_ = 6.9, *τ*_2_ = 2.1; (b) chaotic solution occurs at *τ*_1_ = 9.3, *τ*_2_ = 2.7; (c) the largest Lyapunov exponent diagram of system (55) for variable *τ*_1_ at *τ*_2_ = 2.7; (d) the largest Lyapunov exponent diagram of system (55) for variable *τ*_2_ at *τ*_1_ = 9.3.

However, *E*_∗_ loses stability and the Hopf bifurcation occurs at *τ*_1_ = 6.9, *τ*_2_ = 2.1 in Figure 5(a). When *τ*_1_ = 9.3, *τ*_2_ = 2.7, a chaotic solution occurs in Figure 5(b). To explore the possibility of occurrence of chaos, the largest Lyapunov exponent diagrams are plotted with respect to key parameters *τ*_1_ and *τ*_2_. In Figure 5(c), the largest Lyapunov exponent diagram is plotted for variable *τ*_1_ when *τ*_2_ = 2.7; it is easy to know that when *τ*_1_ > 9.15, the Lyapunov exponent is almost positive; then the chaotic solution occurs. Similarly, in Figure 5(d), the largest Lyapunov exponent diagram is plotted for variable *τ*_2_ when *τ*_1_ = 9.3; it is easy to know that when *τ*_2_ > 2.65, the Lyapunov exponent is almost positive; then the chaotic solution occurs.

## 5. Global Continuation of the Local Hopf Bifurcation

In this section, we will study the global continuation of periodic solutions bifurcating from the point *E*_∗_ for *τ*_1_ is fixed in the interval (0, *τ*_1_0__). Further, the method we used here is based on the global Hopf bifurcating theorem for general functional differential equations introduced by Wu [14]. For convenience, we denote *τ* = *τ*_2_ and write system (3) in the following form:

(56) $$\begin{array}{c} \dot{z}\left(t\right)=F\left(z_{t},\tau ,p\right), \end{array}$$

where *z*_*t*_ = (*z*_1*t*_(*θ*), *z*_2*t*_(*θ*))^*T*^ = (*z*_1_(*t* + *θ*), *z*_2_(*t* + *θ*))^*T*^ ∈ *C*([−*τ*, 0], *R* ^2^). Following the work of Wu [14], we define

  
*X* = *C*([−*τ*, 0], *R* ^2^),
  Σ = *Cl*{(*z*(*t*), *τ*, *p*) ∈ *X* × *R* ^+^ × *R* ^+^, *z*(*t*) is a *p*-periodic solution of (56)},
  
$N=\left\{\left(\overset{-}{z},\tau ,p\right),F\left(\overset{-}{z},\tau ,p\right)=0\right\}$.

**Lemma 7**

Assume that $\left(\overset{-}{z},\tau ,p\right)$ is an isolated center satisfying (*A*1–*A*4) in [14]. Denote by $l_{\left(\overset{-}{z},\tau ,p\right)}$ the connected component of $\left(\overset{-}{z},\tau ,p\right)$ in Σ. Then either

- $l_{\left(\overset{-}{z},\tau ,p\right)}$ is unbounded or
- $l_{\left(\overset{-}{z},\tau ,p\right)}$ is bounded, $l_{\left(\overset{-}{z},\tau ,p\right)}\cap N$ is finite, and $\sum_{(\overset{-}{z},\tau ,p)\in l_{\left(\overset{-}{z},\tau ,p\right)}{⋂}_{}N}\gamma_{m}(\overset{-}{z},\tau ,p)=0$ for all *m* = 1,2, 3,…, where $\gamma_{m}(\overset{-}{z},\tau ,p)$ is the *mth* crossing number of $\left(\overset{-}{z},\tau ,p\right)$, if $m\in J(\overset{-}{z},\tau ,p)$, or it is zero otherwise.

It is well known that if (ii) of the theorem is not true, then $l_{\left(\overset{-}{z},\tau ,p\right)}$ is unbounded. However, when the projections of $l_{\left(\overset{-}{z},\tau ,p\right)}$ onto *z*-space and onto *p*-space are bounded, then the projection of $l_{\left(\overset{-}{z},\tau ,p\right)}$ onto *τ*-space is unbounded. Further, we show that the projection of $l_{\left(\overset{-}{z},\tau ,p\right)}$ onto *τ*-space is away from zero; then the projection of *τ*-space must include [*τ*, *∞*). Following this idea, we can prove our results on the global continuation of the local Hopf bifurcation.

**Lemma 8**

If ((*H* _2_)) and *B* + *E* > 0 hold, nontrivial periodic solutions of (3) are uniformly bounded.

**Proof**

Let *x*(*t*), *y*(*t*) be a nontrivial solution of system (3) through (*φ*, *ψ*) at *t* = 0 with *φ*(0) > 0, *ψ*(0) > 0. Then it follows from (3) that

(57) x(t)=x(0)exp⁡∫0t(r1−r1x(s−τ1)K  −a(1−m)x(s)y(s))ds,y(t)=y(0)exp⁡∫0t(r2−r2y(s−τ)γ(1−m)x(s−τ))ds,

which implies that solutions of system (3) cannot cross the* x*-axes and* y*-axes. Thus, the nontrivial periodic orbits must be located in the interior of the first quadrant.

Since (*x*(*t*), *y*(*t*)) is a nontrivial solution of (3) with *x*(*t*) > 0, *y*(*t*) > 0, then we have

(58) $$\begin{array}{c} \dot{x}\left(t\right)<r_{1}x\left(t\right)\left(1-\frac{x\left(t-\tau_{1}\right)}{K}\right). \end{array}$$

It is easy to know *x*(*t*) < *x*(*t* − *τ*_1_)*e* ^*r*_1_*τ*_1_^ for *t* > *τ*_1_; then if *t* > *τ*_1_, we obtain

(59) $$\begin{array}{c} \dot{x}\left(t\right)<r_{1}x\left(t\right)\left(1-\frac{x\left(t\right)e^{-r_{1}\tau_{1}}}{K}\right), \end{array}$$

which implies that

(60) $$\begin{array}{c} \lim\underset{t\to \infty }{\sup}x\left(t\right)\leq Ke^{r_{1}\tau_{1}}. \end{array}$$

Thus, for any *ε* > 0, there exists a *T* > 0 such that when *t* > *T*, we have

(61) $$\begin{array}{c} x\left(t\right)\leq Ke^{r_{1}\tau_{1}}+\varepsilon :=\delta . \end{array}$$

It follows from the second equation of (3) that, for *t* > *T* + *τ*,

(62) $$\begin{array}{c} \dot{y}\left(t\right)<r_{2}y\left(t\right)\left[1-\frac{y\left(t-\tau \right)}{\gamma \left(1-m\right)\delta }\right]. \end{array}$$

Clearly, *y*(*t*) < *γ*(1 − *m*)*δe* ^*r*_1_*τ*^ for sufficiently large *t*. Thus, the nontrivial periodic solutions lying in the first quadrant of system (3) must be uniformly bounded.

**Lemma**

If ((*H* _2_)) and *B* + *E* > 0 hold, system (3) has no nontrivial periodic solutions with period *τ*.

**Proof**

Assume that system (3) has a nontrivial periodic solution of period *τ*; then the differential system

(63) x˙(t)=r1x(t)(1−x(t−τ1)K)−a(1−m)x(t)y(t),y˙(t)=r2y(t)(1−y(t)γ(1−m)x(t)),

has periodic solution with period *τ*. Due to Lemma 7, we restrict our attention to 0 < *x*(*t*) < *K*, 0 < *y*(*t*) < *γK*(1 − *m*), respectively. System (63) also has the equilibrium *E*_∗_ = (*x*_∗_, *y*_∗_); we define

(64) $$\begin{array}{c} V\left(x,y\right)=\ln\frac{x}{x_{*}}+\frac{x_{*}}{x}+\frac{ax_{*}\gamma {\left(1-m\right)}^{2}}{r_{2}}\left(\ln\frac{y}{y_{*}}+\frac{y_{*}}{y}\right). \end{array}$$

Obviously, *V*(*x*, *y*) is well defined and continuous for all *x*(*t*) > 0, *y*(*t*) > 0. The function *V*(*x*, *y*) satisfies

(65) $$\begin{array}{c} \frac{\partial V}{\partial x}=\frac{1}{x}\left(1-\frac{x_{*}}{x}\right),\;\;\frac{\partial V}{\partial y}=\frac{ax_{*}\gamma {\left(1-m\right)}^{2}}{r_{2}y}\left(1-\frac{y_{*}}{y}\right). \end{array}$$

Equation (65) shows that the positive equilibrium (*x*_∗_, *y*_∗_) is the only extremum of the function *V*(*x*, *y*) in the first quadrant. It is easy to see that the point (*x*_∗_, *y*_∗_) is a minimum, since

(66) lim⁡x→0V(x,y)=lim⁡y→0V(x,y)=lim⁡x→∞V(x,y)=lim⁡y→∞V(x,y)=+∞.

Clearly, the positive equilibrium (*x*_∗_, *y*_∗_) is the global minimum; that is,

(67) $$\begin{array}{c} V\left(x,y\right)>V\left(x_{*},y_{*}\right)=1+\frac{ax_{*}\gamma {\left(1-m\right)}^{2}}{r_{2}}>0 \end{array}$$

holds for all *x*(*t*) > 0, *y*(*t*) > 0.

Calculate the derivative of *V* along the solution of system (3). Use Razumikhin's theorem (see [15]); when 0 < *x*(*t* − *τ*_1_) < *x*(*t*), we have

(68) $$\begin{array}{c} \frac{dV}{dt}<-\frac{r_{1}}{Kx}{\left(x(t-\tau_{1})-x_{*}\right)}^{2}-\frac{a\left(1-m\right)}{y}{\left(y-y_{*}\right)}^{2}<0. \end{array}$$

Thus, *V*(*x*, *y*) satisfies Lyapunov's asymptotic stability theorem; we conclude that

(69) $$\begin{array}{c} \underset{t\to \infty }{\lim}\left(x\left(t\right),y\left(t\right)\right)=\left(x_{*},y_{*}\right), \end{array}$$

which contradicts the fact that system (63) has periodic solutions. This ends the proof.

**Theorem**

If ((*H* _2_)) and *B* + *E* > 0 hold, let *ω*_0_ and *τ*_2_*j*__′  (*j* = 0,1, 2,…) be defined in Case 5 in Section 2. Then, for each *τ* > *τ*_2_*j*__′  (*j* ≥ 1), system (3) has at least *j* + 1 periodic solutions.

**Proof**

It is easy to know that the characteristic matrix of system (3) at the positive equilibrium z* is of the form

(70) $$\begin{array}{c} \Delta \left(z^{*},\tau ,p\right)\left(\lambda \right)=\left(\begin{array}{cc} \lambda -\alpha_{2}e^{-\lambda \tau_{1}} & -\alpha_{1}\\ -\alpha_{5}e^{-\lambda \tau } & \lambda -\alpha_{6}e^{-\lambda \tau } \end{array}\right). \end{array}$$

From the discussion of Section 2, it can be verified that (z*, *τ*_2_*j*__′, 2*π*/*ω*_0_), *j* = 1,2,…, are isolated centers.

Let

(71) $$\begin{array}{c} \Omega_{\epsilon ,2\pi /\omega_{0}}=\left\{\left(\eta ,p\right):0<\eta <\epsilon ,\left|p-\frac{2\pi }{\omega_{0}}\right|<\epsilon \right\}. \end{array}$$

Clearly, if |*τ* − *τ*_2_*j*__′| ≤ *δ* and (*η*, *p*)∈∂*Ω*_*ϵ*_, then the necessary and sufficient conditions for det⁡(Δ(z*, *τ*, *p*)(*η* + *i*(2*π*/*p*))) = 0 are *η* = 0, *τ* = *τ*_2_*j*__′, and *p* = 2*π*/*ω*_0_.

Defining

(72) H±(z∗,τ2j′,2πω0)(η,p)  =det⁡(Δ(z∗,τ2j′±δ,p)(η+i2πp)),

then we have the transversal number

(73) γ(z∗,τ2j′,2πω0)=deg⁡B(H−(z∗,τ2j′,2πω0),Ωϵ,2π/ω0)−deg⁡B(H+(z∗,τ2j′,2πω0),Ωϵ,2π/ω0)=−1.

By Theorem 3.2 of Wu [14], we conclude that the connected component *l*_(z*,*τ*_2_*j*__′,2*π*/*ω*_0_)_ through (z*, *τ*_2_*j*__′, 2*π*/*ω*_0_) in Σ is nonempty. Meanwhile, we have

(74) $$\begin{array}{c} \underset{{(\overset{-}{z},\tau ,p)\in l}_{\left(z^{*},\tau_{2_{j}}^{\prime },2\pi /\omega_{0}\right)}}{\sum }\gamma \left(\overset{-}{z},\tau ,p\right)<0 \end{array}$$

and hence *l*_(z*,*τ*_2_*j*__′,2*π*/*ω*_0_)_ is unbounded.

From (36), we see that, for *j* ≥ 1, 2*π*/*ω*_0_ < *τ*_2_*j*__′. Then, we are in a position to prove that the projection of *l*_(z*,*τ*′_2_*j*__,2*π*/*ω*_0_)_ onto *τ*-space is $\left[\overset{-}{\tau },\infty \right)$, where $\overset{-}{\tau }<\tau_{2_{j}}^{\prime }$. Clearly, it follows from the proof of Lemma 9 that system (3) with *τ* = 0 has no nontrivial periodic solution. Hence, the projection of *l*_(z*,*τ*_2_*j*__′,2*π*/*ω*_0_)_ onto *τ*-space is away from zero.

For a contradiction, we suppose that the projection of *l*_(z*,*τ*_2_*j*__′,2*π*/*ω*_0_)_ onto *τ*-space is bounded. This means that the projection of *l*_(z*,*τ*_2_*j*__′,2*π*/*ω*_0_)_ onto *τ*-space is included in an interval (0, τ*). Noting that 2*π*/*ω*_0_ < *τ*_2_*j*__′ and applying Lemma 9, we have *p* < τ* for (*z*, *τ*, *p*) belonging to *l*_(z*,*τ*_2_*j*__′,2*π*/*ω*_0_)_. This implies that the projection of the connected component *l*_(z*,*τ*_2_*j*__′,2*π*/*ω*_0_)_ onto *p*-space is bounded. In addition, from Lemma 8, we obtain that the projection of *l*_(z*,*τ*_2_*j*__′,2*π*/*ω*_0_)_ onto *z*-space is bounded if the projection of *l*_(z*,*τ*_2_*j*__′,2*π*/*ω*_0_)_ onto *τ*-space is bounded. Thus, the connected component *l*_(z*,*τ*_2_*j*__′,2*π*/*ω*_0_)_ crossing through (z*, *τ*_2_*j*__′, 2*π*/*ω*_0_) is bounded, which is a contradiction. This implies that the projection of *l*_(z*,*τ*_2_*j*__′,2*π*/*ω*_0_)_ onto *τ*-space is $\left[\overset{-}{\tau },\infty \right)$ for each *j* ≥ 1, where $\overset{-}{\tau }<\tau_{2_{j}}^{\prime }$. This is the end of the proof.

## 6. Conclusions

In this paper, we investigate the effect of the time delays *τ*_1_ and *τ*_2_ on the stability of the positive equilibrium of system (3) and derive the direction and stability of the Hopf bifurcation. Numerical simulations are carried out to illustrate the theoretical prediction and to explore the complex dynamics including chaos. Finally, we study the global continuation of periodic solutions bifurcating from the point *E*_∗_ for *τ*_1_ is fixed in the interval (0, *τ*_1_0__) and show the global existence of the periodic solutions.
